# Biliary Tract Cancer: Current Medical Treatment Strategies

**DOI:** 10.3390/cancers12051237

**Published:** 2020-05-14

**Authors:** Ester Oneda, Mohammed Abu Hilal, Alberto Zaniboni

**Affiliations:** 1 Department of Clinical Oncology, Fondazione Poliambulanza, 25124 Brescia, Italy; alberto.zaniboni@poliambulanza.it; 2 Department of Surgery, Fondazione Poliambulanza, 25124 Brescia, Italy; mohd.abuhilal@poliambulanza.it

**Keywords:** cholangiocarcinoma, gallbladder cancer, chemotherapy, targeted therapy

## Abstract

Background: Biliary tract cancers (BTCs) include cholangiocarcinomas and gallbladder cancers usually present at an advanced stage, which are considered resectable in less than 20% of cases and characterised by poor prognosis. Methods: In this review, we discussed the most recent therapeutic options on the basis of the most updated and complete reviews and recent prospective studies in selected BTC patients. Results: Due to the high recurrence rate of BTCs, we suggest the new recommendations that have been made on adjuvant chemotherapy and radiotherapy treatment after surgery. New chemotherapy combinations in advanced-stage patients allow a better survival benefit than the standard treatment. Furthermore, the revelation of complex molecular events and their interactions and relationships with some risk factors allowed the development of targeted/toxic agents alone or combination with chemotherapy that is really promising. In unresectable patients, hepatic arterial infusion of high-dose chemotherapy or selective internal radiotherapy could offer a primary mass volume reduction or its resection with the maintenance of liver function. Conclusions: The therapeutic landscape for BTCs is blooming again, the knowledge of their biology is still growing, but the available data on chemotherapy, radiotherapy, locoregional treatments, and target therapies have added hopes to improve patient survival.

## 1. Introduction

Tumours arising from the epithelium along the biliary tree are referred to as biliary tract cancers (BTCs), which include cholangiocarcinoma (CCA) and gallbladder cancer. Cholangiocarcinomas (CCAs) can then be differentiated by their anatomical origin into intrahepatic CCA (iCCA) and extrahepatic CCA (eCCA). Lesions involving the second-order bile ducts are defined as iCCA and accounts for 5–10% of all CCAs. In the other hand, eCCAs are divided into perihilar CCAs (pCCAs), when they lie above the cystic duct (CD), accounting for 60–70% of eCCAs, and distal CCAs (dCCAs), when they lie below the CD, accounting for the remaining part [1]. 

Gallbladder cancer is the most frequent biliary tract cancer, comprising 80–90% according to autopsy studies [2,3], followed by CCAs, which also represents the second most common primary liver cancer accounting for nearly 15% of all liver malignancies [4] and for approximately 3% of all gastrointestinal tumours [5]. The American Cancer Society’s estimates a total of 11,980 newly diagnosed cases for approximately 4090 deaths from gallbladder and bile duct cancers in the United States for 2020 [6]. 

BTCs are characterized by poor prognosis, with 5-year relative survival rates ranging from 2% to 15% for iCCAs, 2% to 30% for eCCAs, and 2% to 70% for gallbladder cancer, depending on stage [7]. Similarly, poor mean overall survival (OS) rate for patients with CCAs (less than 24 months) and gallbladder cancer (6 months) has been reported [8].

Cholangiocarcinoma and gallbladder cancers occur largely after the fourth and sixth decade of life, respectively, and are more frequent in men than in women [9]. They are relatively rare in the Western world; however, higher incidence clusters have been found in some areas of Asia and the Andes [7], with the highest incidence rates of iCCAs reported in northern Thailand (113 per 100,000 person-years among men and 50 per 100,000 person-years among women), possibly due to genetic and environmental differences [9]. In fact, endemic fluke Opisthorchis viverrini (a flatworm that infects humans via ingestion of raw or pickled fish) infection in this area has been reported, and CCA makes up 89% of primary liver cancers [9]. 

Other risk factors for CCA are parasitic infections; bile duct disorders, such as primary sclerosing cholangitis (PSC), biliary cysts, or ductal stone disease; toxins; viral hepatitis, such as hepatitis C and B. These risk factors are associated with chronic inflammation of the biliary tree and liver injury that predispose the affected individuals to carcinogenesis [5]. 

Gallbladder cancer, instead, has a particularly high incidence in Chile, Japan, and northern India [2] and is commonly associated with chronic inflammation, with metaplasia progressing to dysplasia, carcinoma in situ, and then, invasive cancer. Gallstone disease, gallbladder polyps, chronic Salmonella infection, congenital biliary cysts, and abnormal pancreaticobiliary duct junction have been reported as important precursors [2]. 

Biliary tract cancers usually present at an advanced stage, with less than 20% of patients considered resectable at presentation [7]. In addition, when possible, adjuvant therapy is usually indicated to improve rates of relapse-free survival (RFS) and overall survival (OS) [7]. In non-operable patients, stent placement can be offered to treat obstructive jaundice, control symptoms such as pruritus, biliary cirrhosis, and cholangitis and decrease related secondary morbidity [10]. Endobiliary techniques have made notable progress, improving stent median patency with self-expanding bare-metal stents (SEMS) [10] and offering local disease control with novel therapies, such as radiofrequency ablation (RFA), photodynamic therapy (PDT), and irreversible electroporation (IRE), thus providing a wider window for administration of cytotoxic therapy to a larger pool of patients [11].

Studies of adjuvant treatment options have historically been small, retrospective, and non-randomized and include a mixture of patients with gallbladder and other biliary tract tumours. Recently, results from prospective trials of adjuvant therapy for BTC have been published and have made a step forward in the current recommendations [7]. In the advance setting, during the past decade, the evidence base for the treatment consisted of gemcitabine–cisplatin as front line regimen [12], but the median overall survival was under 1 year. In order to improve outcomes, novel targeted/toxic agents alone or in combination with chemotherapy are under investigation. 

The purpose of this review is to analyse and discuss the latest therapeutic options, including adjuvant, first- and second-line chemotherapy, targeted therapies, combination treatments, and locoregional therapies, for patients with biliary tract cancers.

In recent years, some studies have focused on BTC behaviour and their prognosis by anatomical site, their responsivity to chemotherapeutic agents and their molecular pathophysiology, finding new treatment options, and carcinogenic role of some genes, and genes affected by copy number alterations which can benefit from targeted therapy. We herein discuss these latest therapeutic strategies and their possible future applications.

## 2. Results

### 2.1. Chemotherapy Treatment 

#### 2.1.1. Adjuvant Therapy 

Three important Phase III randomised clinical trials, comparing chemotherapy treatment to observation alone in resected BTC patients, have been published in the last 2 years [13,14,15]. The BCAT trial and the PRODIGE-12/ACCORD-18 trial have failed to show any benefit with gemcitabine (Gem) or with the combinations of gemcitabine and oxaliplatin (GemOx) chemotherapy in patients with resected BTC when compared to an observation alone arm [13,14]. Unlike the previous two trials, the BILCAP trial [15] have interestingly shown a significant benefit in terms of OS associated with capecitabine (Cap), administered for eight cycles, vs. observation alone (OS 51.1 months, 95% CI 34.6–59.1 vs. 36.4 months, 95% CI 29.7–44.5). Even though the BILCAP study did not meet its primary end-point goal (pre-planned target HR of 0.69), in the ITT population, a significant survival benefit of 53 months associated with Cap vs. 36 months associated with observation alone (HR 0.81 (95% CI 0.63–1.04); *p*-value 0.097) has been reported. In addition, a significant benefit in terms of RFS (24.4 months (95% CI 18.6–35.9) vs. 17.5 months (95% CI 12.0–23.8); HR 0.75 (95% CI 0.58–0.98); *p*-value 0.033) has been noted. These trials were compared in different meta-analyses. and their results are summarised in Table 1. 

A meta-analysis from Messina et al. [19] has shown that adjuvant CT provides a mild benefit in terms of RFS (HR: 0.83, 95% CI 0.69–0.99) and effect on OS, but not in the overall population (HR: 0.91, 95% CI 0.75–1.09) nor the node positive subgroup (HR: 0.84, 95% CI 0.65–1.08), despite a higher risk of ≥ G3–G4 adverse events (AEs; RR: 3.03, 95% CI 2.22–4.15). In contrast, Caparica et al. [20], analyzing those three clinical trials, stated that no difference in OS was observed between chemotherapy and observation, whereas a significant improvement was seen in RFS. Furthermore, a trend in benefit from adjuvant chemotherapy was observed in N+ patients. However, the lack of individual patient data, long recruitment periods, differences in clinical trial design, heterogeneity in CT regimens and patient’s characteristics, different access to chemotherapy delivered after disease recurrence, and no stratification of patients according to BTC subtype could have skewed the results [19]. Lamarca et al. [21] found other limitations in the design of these studies, such as follow-up time, power, and data maturity.

Based on these findings, the ASCO guidelines have [7] recommended adjuvant capecitabine for a period of six months following curative resection of BTCs (CCA and GBC). Researchers agree on the fact that 12–16 weeks of recovery time is required after surgery prior to starting adjuvant therapy, and the administration of chemotherapy during a limited period of time (rather than until disease recurrence) is accepted as standard practice in the adjuvant setting and has been shown to be safe. Finally, based on the data available, chemotherapy based on fluoropyrimidines seems to be preferable [22].

This was also confirmed by the Japanese Phase II randomized KHBO 1208 trial [16] that compared 6-months of CT with biweekly Gem to tegafur/gimeracil/oteracil (S-1) in BTC Asian patients after major hepatectomy. S-1 has shown superior outcomes (*p* < 0.10) in terms of OS and 2-year RFS. Nowadays, the Phase III Japanese ASCOT trial is testing S-1 vs. observation in patients with resected BTC [17], while the multinational ACTICCA-01 trial [18] is testing cisplatin plus gemcitabine (the CisGem regimen), which is the current standard of care in patients with advanced disease and in patients with curatively resectable disease (Table 1 shows their regimen schedules). Moreover, the trial also plans to test adjuvant radiotherapy after R1 resection (and no disease progression after adjuvant chemotherapy). 

#### 2.1.2. Neoadjuvant Therapy

Neoadjuvant therapy could allow us to treat more patients than adjuvant therapy; this suggestion is mainly based on the observation that only 55% of patients who received capecitabine in the BILCAP trial have completed the planned eight cycles of treatment. However, neoadjuvant chemotherapy administration is not always possible due to the patient symptoms such as jaundice or other morbidities that could hold back treatment administration [23]. Unfortunately, no studies have been published on this specific area. 

#### 2.1.3. Advanced Disease: First-Line Treatment 

The milestone of first-line treatment for BTC comes from the ABC-02 study [12] that suggested the efficacy of gemcitabine–cisplatin (GemCis) chemotherapy. Although this regimen has remained the standard of care, the median overall survival is still reported at just under 1 year [24]. Shroff et al. [25] demonstrated that the addition of nab-paclitaxel to standard doublet therapy (known as the GAP regimen: gemcitabine, nab-paclitaxel, and cisplatin) has improved survival. Notably, owing to poor tolerability observed, Gem and nab-paclitaxel doses were reduced by 20% from the standard dose. Interestingly, 12 patients were converted to resectable disease and completed surgery, 2 of whom achieved a pathologically complete response (PCR). Only 16% of patients withdrew owing to adverse effects, despite the high rate (58%) of grade 3 or higher adverse events [24,25]. The reduced-dose triplet is being evaluated against GemCis in the ongoing Phase III SWOG 1815 trial (NCT03768414). 

The superiority of the combination regimen of folinic acid, fluoropyrimidine, irinotecan, and oxaliplatin (FOLFIRINOX) over Gem in pancreatic cancer patients [26] has led to the rationale for using this regimen in BTC patients. In a small retrospective series, FOLFIRINOX, as first-line treatment for BTC, has led to a disease control rate of 75% and OS of 15 months [27,28]. The ongoing Phase II/III PRODIGE38-AMEBICA trial (NCT02591030) aims to demonstrate an improvement in OS of 4 months in favour of the modified FOLFIRINOX (no 5-FU bolus on day 1) vs. GemCis [29]. The NIFE Phase II trial aims to challenge the current palliative first-line therapy for BTC by the use of nanoliposomalirinotecan/5-FU/leucovorin (nal-IRI). This trial is based on the assumption that ≥60% of patients will be progression-free after 4 months of nal-IRI [30]. In Table 2, first-line treatments are compared.

#### 2.1.4. Advanced Disease: Beyond the First Line 

After first-line therapy, 25%–50% of patients who progressed were still able to receive second-line chemotherapy (CT2) [31]. Discordant opinions have been suggested regarding the potential benefit of CT2 with a marginal activity (RR 7.2%, DCR 49.5%), and limited efficacy in unselected populations (OS 7.2 months, PFS 3.2 months). The ABC-06 Phase III trial demonstrated OS benefit with the modified combination of folinic acid, fluorouracil and oxaliplatin (mFOLFOX6) plus active symptom control (ASC) vs. the effect of ASC alone, in patients who had progressed to first-line GemCis. Notably, the effect of mFOLFOX6 was maintained regardless of previous platinum sensitivity, and 20% of patients experienced disease control of at least 9 months [21]. However, the study did not compare mFOLFOX6 regimen with a fluoropyrimidine alone arm, so it was not clear if the combination therapy was more efficient than monotherapy in this setting [32]. CT2 with the combination of mitomycin C and capecitabine vs. capecitabine alone yielded poor outcomes; oxaliplatin plus irinotecan (XELIRI) achieved PFS and OS benefit over irinotecan monotherapy [33] in a Chinese population the combination of folinic acid, fluorouracil and irinotecan (FOLFIRI) plus bevacizumab [34] and the sequence Gemox-FOLFIRI achieved 21.9 months OS in a small patient series [35]. In addition, trifluridine/tipiracil (FTD/TPI) was tested in BTC patients in II-line; as expect, no objective response (OR) was seen [36], although 60% of patients had been treated with up to three chemotherapy lines. A Phase II single-arm trial evaluating the combination of FTD/TPI and irinotecan for patients with refractory BTC is ongoing (NCT04072445), supporting the idea that this combination will likely have a higher response rate. The meta-analysis from Ying and Chen [37] has indicated that 1-year OS was 21.1% for a single targeted agent, 29.6% for a single toxic agent (mainly fluoropyrimidine alone), 26.9% for gemcitabine-based combination therapy, and 23.3% for taxanes-based combination therapy; in fact, in all cases, it was higher than the 15% observed in fluoropyrimidine-based combination therapy. However, no head-to-head comparison data were available for combination therapy vs. monotherapy [37].

These studies (summarised in Table 3) have shown heterogeneous results which emphasises the need of an appropriate selection of patients. Many single institutional studies have tried to define some prognostic tools for selecting patients who could benefit from CT2, such as age, performance status, surgery for primary tumour, a good response to first-line therapy, PFS longer than 6 months, neutrophil-to-lymphocyte ratio, and carcinoembryonic antigen, CA19-9, leucocyte, cholinesterase and albumin concentrations [32]. Only one multicentre European study has developed and validated (in three countries) a prognostic model based on four independent prognostic factors: the baseline performance status, the reason of ending the first line-therapy, surgical treatment for the primary tumour, and the presence of peritoneal carcinomatosis [32]. 

### 2.2. Radiotherapy

As stated by European Society of Medical Oncology(ESMO) guidelines, radiotherapy may be considered for certain patients, but there is limited clinical evidence for its effectiveness; therefore they are not currently commonly used in Europe outside of clinical trials. In particular, radiotherapy can be used to relieve some symptoms of biliary tract cancer-like pain and other symptoms by shrinking tumours that block blood vessels or bile ducts, or press on nerves [38]. A retrospective analysis of inoperable iCCA patients treated with definitive radiation therapy suggested that a biologically effective dose (BED) >80.5 Gy seems to be ablative, with long-term survival rates that compare favorably to resection [39]. The metanalysis of Ren et al. showed a 5-year OS benefit for eCCA and gallbladder cancer patients treated with adjuvant radiotherapy than without, with a major benefit in for those with lymph node-positive disease (OR = 0.15; 95% CI 0.07–0.35; *p* < 0.00001) and margin-positive disease (OR = 0.40; 95% CI 0.19–0.85; *p* = 0.02) [40]. Manterola et al. [41] have completed a literature review on the combination of chemo-radiotherapy (CRT) with fluoropyrimidines as a radio-sensitizer in the adjuvant setting or radiotherapy (RT) alone for resected gallbladder cancer. The effectiveness of adjuvant CRT continues to be controversial, although, in the SWOG080 trial, adjuvant CT with gemcitabine plus capecitabine (Gem-cape) followed by concomitant chemoradiation resulted in an impressive 2-year OS rate of 65% (95% CI, 53%–74%) among 79 patients with resected BTC, independently of the resection margin status [42]. Unfortunately, the paucity and low quality of data, including the lack of direct comparisons, did not permit a consensus on the modality of radiation, dose, or the adequate time for administration. It could reasonably be assumed that CRT could be more beneficial on OS than other adjuvant treatments with less-toxic effects due to the low radiation doses used [42]. 

However, based on these findings the ASCO guidelines have [7], the role of chemo-radiotherapy remains unclear and this suggests that its use should be limited to patients with eCCAs with R1 resection or other high-risk factors.

### 2.3. Targeted Therapy

Early studies on the genetic pathophysiology of CCAs focused on the elucidation of the carcinogenic role of single genes, often found mutated in other malignancies, or genes affected by copy number alterations [43]. In recent years, due to the advent of next-generation sequencing technology, a series of complex molecular events, their interactions, and their relationships with risk factors and causative events were revealed in biliary cancers. Among them, mutations in the genes of isocitrate dehydrogenases (IDH, isoforms 1 and 2), fusions of the fibroblast growth factor receptor 2 (FGFR2), and mutations of chromatin-remodeling genes, such as AT-rich interaction domain 1A (ARID1A), protein poly-bromo1 (PBRM1), and BRCA1-associated protein 1 (BAP1), were detected [8]. Target treatments were extensively discussed in very recent reviews [8,31,43]. 

Isocitrate dehydrogenase (IDH, isoforms 1 and 2) is involved in glucose metabolism, and mutation of these genes leads to the accumulation of the oncometabolite D-2hydroxyglutarate (2-HG) and resultant epigenetic dysregulation and aberrant cell signalling. Mutations in IDH1 are found in 10%–25% of patients with advanced CCAs [44]. Ivosidenib (AG-120), an oral inhibitor of IDH1, was administered to patients with mutated advanced CCAs who were ineligible for resection and had disease progression following 1 to 2 prior systemic therapies [44]. Ivosidenib prolonged median PFS compared to the effect of placebo (2.7 vs. 1.4 months, HR 0.37; *p* < 0.001), there was a numerical improvement in median OS (10.8 vs. 9.7 months, HR 0.69; *p* = 0.06), and DCR nearly doubled (53% vs. 28% for placebo) [44]. Other IDH selective inhibitors, such as IDH305 (IDH1 inhibitor), enasidenib (IDH2 inhibitor), and AG-881 (IDH1 and 2 inhibitor), are still under study [8,45]. 

FGFR2 gene rearrangements code for constitutively activated fusion products, resulting in aberrant mitogenic signalling with dysregulation of cell proliferation, survival, and angiogenesis. FGFR2 alterations have been reported in 13% to 17% of patients with CCAs [31,43]. Multiple oral tyrosine kinase inhibitors (TKIs) targeting FGFR were tested in patients with advanced CCAs, who presented FGFR gene fusions or other alterations and progressed after prior first-line therapy. Infigratinib (BGJ398) [46], pemigatinib (INCB054828) [47], derazantinib (ARQ 087) [48], and TAS-120 [49] demonstrated ORRs of 31%, 36%, 21%, and 25%, respectively, with a median PFS of 6.8 months for infigratinib, 6.9 months for pemigatinib, 5.7 months for derazantinib (data from the study with TAS-120 are not yet mature), an OS of 12.5 months for infigratinib and 21.1 months for pemigatinib (for Derezantinib and TAS-120, OS is not yet reached), and a DCR of approximately 80%. The ongoing Phase III trials will compare some of these TKIs to GemCis for first-line treatment of advanced CCAs [31,43]. 

HER2 (or ErbB2) alterations are found in 5.7% to 7.6% of all CCAs, and in 10%–32% of iCCAs [8,31]. The combination of trastuzumab and pertuzumab in the MyPathway trial (NCT02091141) [47], resulted in disease control in six out of eight HER2-amplified/overexpressed patients with a BTC, while two of the nine patients responded to neratinib in the SUMMIT trial (NCT01953926) [50]. Safety and activity were shown with the panHER inhibitor varlitinib combined with CT (RR 27%, DCR 70%) [51], while ongoing trials are evaluating afatinib (NCT02451553) and trastuzumab–emtansine (NCT02999672) [31]. 

VEGF was found to be overexpressed in 53.8% of iCCAs and 59.2% of eCCAs [43]; different associations between bevacizumab and CT and multikinase inhibitors, such as vandetanib and regorafenib [52], were tested and have shown some activity; ramucirumab is also currently under investigation [8,31,43]. 

Activation of missense BRAF mutations is reported in 11%–22% of CCAs, depending on the studies’ population [53,54]. Vemurafenib, in combination with an MEK inhibitor, appeared to be promising in cancers harboring BRAF V600 mutations [31]. Selumetinib, an inhibitor of MEK1/2 proteins, supported stable disease alone or in combination with chemotherapy. An ongoing Phase II trial is evaluating the combination of solumetinib with gemcitabine vs. gemcitabine alone. Furthermore, other promising MEK inhibitors, including refametinib, trametinib, and MEK162, are under investigation in several clinical trials [8].

Unsatisfactory results derived from the use of MET inhibitors such as cabozantinib (abnormal MET activation has been found in 12%–58% of iCCAs) PI3K/AKT/mTOR inhibitors showed exiguous responses despite 12.5% of gallbladder cancers displaying activated mutations of PIK3CA. The ongoing Phase II STARTRK-2 trial is investigating entrectinib in patients with solid tumours harboring NTRK, ROS1, or ALK gene rearrangements/fusions, including those with CCAs [55]. 

Hypermutated BTCs characterized by increased expression of PD1/PD-L1 have a poor prognosis. Less than 1% of patients had high microsatellite instability (MSI), and the trials with immune-checkpoint inhibitors showed infrequent responses in the microsatellite stable tumours [31]. Immunotherapy was tested in BTC patients with some partial response and disease control, with a 12-month OS of 33% [32]. 

In all subtypes, clinical studies with PD-1 and PD-L1 blockade agents have shown ORRs in the 5%–20% range and meaningful disease control [56,57,58,59,60,61]. Unfortunately, at the moment, PD-L1 expression, MSI, mismatch repair deficiencies, and tumour mutational burden are not available as predictive biomarkers [32]. To improve the immune response, the combination of CT (TOPAZ-1, IMMUCHEC, NCT03111732, NCT03101566, NCT04027764, NCT03982680, NCT04003636), RT (CORRECT trial), locoregional procedures (NCT02821754), targeted agents, and new immunotherapy effectors are under investigation [31,62]. 

Promising targets for potentially interesting inhibitors are the proteins of the JAK/STAT, Wnt/β-catenin, Hedgehog and Notch signalling pathways, inhibitors that target mutations in chromatin-remodelling genes, such as ARID1A, PBRM1, and BAP1, and inhibitors of cancer-associated fibroblasts [8]. New drugs, aimed at simultaneously targeting several cell types that are critically involved in the development and progression of CCA, are in development.

A curious observational study, published in JAMA Oncology, showed that the use of aspirin in patients with BTC was associated with a decreased risk of death (HR of 0.63 for gallbladder cancer and of 0.71 for CCA) which may be due to the drug’s inhibition of platelet aggregation slowing the spread of metastatic cancer cells. The researchers noted that incidental aspirin users who had no prior history of using the drug had a greater survival benefit from postdiagnosis aspirin use than those who were prevalent users, but they did not have an explanation [63]. One hypothesis is that tumours that developed during aspirin consumption were less susceptible to its beneficial function. 

### 2.4. Locoregional Treatments

Considering that most patients present with unresectable advanced disease confined to the liver at diagnosis, liver-directed therapy could offer disease control or, even better, a primary mass volume reduction with the maintenance of adequate liver function. The liver’s dual blood supply preferentially delivers high doses of chemotherapeutic agents to the hepatic artery, which supplies nearly all of the tumour’s blood flow, while blood delivered by the portal vein maintains the health of the non-neoplastic liver parenchyma. Because the liver clears the chemotherapeutic agent via first-pass metabolism, locoregional approaches could diminish the systemic toxic effects of chemotherapy. A single-institution, Phase II clinical trial [64] investigated hepatic arterial infusion (HAI) of high-dose chemotherapy combined with first-line systemic chemotherapy in patients with unresectable intrahepatic CCA. Via HAI, high-dose floxuridine, a precursor of fluorouracil chemotherapy, was delivered directly into the liver, while systemic GemOx chemotherapy was administered. A response rate of 58% and an excellent disease control rate of 84% in the primary tumour at 6 months was achieved. Four patients underwent resection, and 1 patient had a complete pathologic response. The median PFS was 11.8 months and the median OS was 25.0 months, with a 1-year OS rate of 89.5%. A response in the dominant liver mass helped to prevent liver-related complications, such as biliary obstruction and portal vein occlusion, which are often associated with interruption of systemic therapy. This finding suggests that longer survival was correlated with the response of the primary tumour. Interestingly the subgroup analysis showed significant improvement in survival in patients with IDH1/2 mutated tumours (2-year OS 90%, 95% CI, 73%–99%) vs. wild-type tumours (2-year OS 33%, 95% CI, 18%–63%) [64]. 

In another Phase II study, the yttrium-90 microspheres (MISPHEC) trial [65], the technique of radioembolization, also known as selective internal radiotherapy (SIRT), was used in locally advanced iCCA in combination with systemic therapy with the GemCis regimen. Radiolabeled microspheres were administered via the hepatic arteries, delivering radiotherapy when reaching the tumour vasculature. The results showed PFS of 14 months and a median OS of 22 months, with an increased number of downstaged tumours and chance of surgery [41]. In the SIRCCA phase II trial, SIRT followed by CIS–GEM chemotherapy vs. CIS–GEM chemotherapy alone as first-line treatment of patients with unresectable intrahepatic CCA is currently randomising patients with unresectable CCA to either chemotherapy alone or resin-microsphere SIRT followed by chemotherapy, and a study comparing SIRT with chemoembolization is ongoing [66].

## 3. Discussion

BTCs are infrequent cancers that have been poorly studied in the past decades and have few treatment options, low response rates, and bad prognosis. Their rarity and the difficulty of obtaining good diagnostic samples make the effective development of translational research in this field significantly difficult. One of the most important steps forward will be the definition of the true value of some of the risk factors (such as R1 ore node positivity), and to correctly use them as stratification factors to pre-plan a correct statistical analysis [22] and for tailored treatment strategies. Moreover, valid and specific biomarkers are needed to select patients who are more likely to benefit from specific adjuvant strategies. Last but not least, there is a need for in-depth translational research to understand the mechanisms of resistance and disease recurrence. In this regard, an appropriate study design is crucial, where new Phase III randomised studies, comparing experimental arms with an active (non-observational) control arm (currently: capecitabine), need to be initiated after careful thoughts. 

Although complete surgical resection (R0) remains the most effective approach for prolonging survival, disease recurrence is not uncommon. Furthermore, even if surgery could be technically performed, this could not always be contemplated due to patient comorbidities. The development of interventional techniques with lower morbidity is therefore essential to prolong survival in that population. Locoregional therapies should offer an important next step to improve survival by controlling local disease progression. As a result, some groups are experimenting with modifications to photoactive compounds in PDT, probe modifications in RFA, and combinations of locoregional therapy and concurrent chemotherapy [4]. Some progress has also been made in first-line therapies. The triple therapy with gemcitabine–cisplatin plus nab-paclitaxel resulted in a higher median PFS, OS, and RR than the combination therapies reported previously [24]. Other combinations are under investigation and have shown promising results. 

Moreover, after tumour progression, there is no clear evidence of whether a combination therapy can be superior to a single targeted/toxic agent. However, since the overall quantity and quality of data regarding second-line therapy is poor and considering the risk of bias in comparisons between the observation studies, prospective randomized studies are strongly recommended [37]. Due to the rarity of advanced BTC patients, future multi-institutional clinical trials should promote good-sized studies that focus on stratifying patients on the basis of their anatomic subtype and genetic drivers in order to predict response to the new therapeutic options and the prognosis. 

Despite the considerable progress that has been made in molecularly profiling BTCs, knowledge on the cause-and-effect relationship between the molecular changes and transformation of normal biliary epithelium to invasive malignancy, and our understanding of the interactions among the various signalling pathways and components that produce the cancer cell phenotypes, are still lacking. 

At the top of the list is the definition of some prognostic tools for selecting patients who could benefit more from adjuvant chemotherapy or radiotherapy or the combination of both. For example, we need some lines about which therapy is preferred based on the volume, extension of disease, the presence of jaundice or other symptoms, age, performance status, serum carcinoembryonic antigen and CA19-9 levels and liver function. In addition, for the selection of patients to be candidates for a first-line treatment, we must evaluate the recurrence time, the primary surgery, the extent and locations of the metastatic disease and whether it may be susceptible to locoregional therapies, comorbidities and life expectancies. Unfortunately, no studies have yet established clear selection criteria and we hope this will be the topic of the next work.

## 4. Conclusions

The therapeutic landscape in BTC has expanded considerably in recent years after being nearly forgotten for a few decades. Combined therapy with chemotherapy, locoregional treatment and immunotherapy are promising strategies. The knowledge of the biology of BTC is still limited compared to that of other solid cancers, but the available data on target therapies have added hopes for the future management of hepatobiliary cancers and will likely continue to improve patient outcomes. Results from ongoing clinical trials are eagerly awaited to further elucidate the optimal management of this aggressive malignancy.

## Figures and Tables

**Table 1:** Summary of adjuvant treatments.

Study	Number and Type of Patients	Study Arms	RFS	OS
**Primrose et al. [15] randomised, controlled, multicentre, phase III BILICAP trial**	447 CCA or muscle-invasive gallbladder cancer	Cap: 1250 mg/m^2^ twice a day on days 1 to 14 of a 3-week cycle for 24 weeks (8cycles) vs. Obs	Cap: 24.6 months (95% CI, 18.9 to 36.7 months) Obs: 17.6 months (95% CI, 12.8 to 27.6 months) HR 0.76 (95% CI, 0.58 to 0.99; *p* = 0.039)	Cap: 51.1 months (95% CI, 34.6 to 59.1 months) Obs: 36.4 months (95% CI, 29.7 to 44.5 months) HR: 0.81 (95% CI, 0.63 to 1.04; *p* = 0.097)
**Edeline et al. [14] multicentre, open-label, randomised phase III PRODIGE 12-ACCORD 18 trial**	196 resected BTC	Gem 1000 mg/m^2^ on day 1 and oxaliplatin 85 mg/m^2^ on day 2 for 12 cycles vs. Surveillance	GemOx: 30.4 months (95% CI, 15.4 to 43.0 months) Surveillance: 18.5 months (95% CI, 12.6 to 38.2 months) HR 0.71 (95% CI, 0.62 to 1.25; *p* = 0.48)	GemOx: 24-months OS 69% 48-months OS 51% 72-months OS 51% Surveillance: 24-months OS 76% 48-months OS 52% 72-months OS 48%
**Ebata et al. [13] randomised phase III BCAT trial**	225 resected BTC	Gem 1000 mg/m^2^ on days 1,8, and 15 every 4 weeks (6 cycles) vs. Obs	Gem: 36.0 months Obs: 39.9 months HR 0.93 (95% CI, 0.66 to 1.32; *p* = 0.693)	Gem: 62.3 months Obs: 63.8 months HR 1.01 (95% CI, 0.70 to 1.45; *p* = 0.964)
**Kobayashi et al. [16] multicentre, randomised phase II KHBO 1208 trial**	70 BTC after major hepatectomy	Gem 1000 mg/m^2^ on days 1 and 8 every 2 weeks vs. S1 80 mg/m^2^/day for 28 days every 6 weeks	1-year 51.4% vs. 62.9% 2-year 31.4% vs. 51.4%	1-year 80% vs. 97.1% HR 0.48 (CI 90%, 0.24-0.93) 2-year 60% vs. 80%
**Nakachi et al. [17] open-label, multicentre, randomised phase III ASCOT trial**	350 resected BTC	S1: 40–60 mg/day related to BSA (body surface area) vs. Obs	n.a	3-year OS n.a vs. 47%
**Stein et al. [18] randomised, multidisciplinary, multinational phase III ACTICCA-01 trial**	CCA and gallbladder cancer	Cis/Gem on days 1 and 8 every 3 weeks, Cisplatin (25 mg/m^2^) and Gemcitabine (1000 mg/m^2^) vs. Cap from days 1 to 14 every 3 weeks (1250 mg/m^2^, twice daily)	n.a	n.a

CCA = cholangiocarcinoma; BTC = biliary tract cancer; Gem = gemcitabine; Cap = capecitabine; Obs = observation; S1 = tegafur/gimeracil/oteracil; Cis = cisplatin; OS = overall survival; RFS = relapse-free survival; n.a = not available; DFS = disease free survival; CI = confidence interval.

**Table 2:** Summary of first-line treatments in advanced settings.

	Fist Line			
Study	Number and Type of Patients	Study Arms	PFS	OS
**Valle et al. [12] Phase III ABC-02 trial**	410 locally advanced or metastatic CCA, gallbladder cancer, or ampullary cancer	GemCis: cisplatin 25 mg/m^2^ followed by Gem 1000 mg/m^2^ (on days 1 and 8, every 3 weeks for 8 cycles) vs. Gem alone 1000 mg/m^2^ (on days 1, 8, and 15, every 4 weeks for six cycles) for up to 24 weeks.	8.0 vs. 5.0 months	11.7 vs. 8.1 months HR (0.64; 95% confidence interval, 0.52 to 0.80; *p* < 0.001)
**Shroff et al. [25] open-label, single-arm, Phase II trial**	60 advance BTC	GAP: Gem 1000 mg/m^2^, cisplatin, 25 mg/m^2^, and nab-paclitaxel, 125 mg/m^2^, on days 1 and 8 of 21-day cycles	11.8 months (95% CI, 6.0 to 15.6)	19.2 months (95% CI, 13.2 months to not estimable)
**Phelip et al. [29] Phase II/III trial PRODIGE38-AMEBICA trial**	intra or extra hepatic or hilar or gallbladder carcinoma	FOLFIRINOXm Oxali 85 mg/m2, IRI 180 mg/m^2^ (IV 90 min), folinic acid 400 mg/m^2^ (IV 2 h), 5 FU 2400 mg/m^2^ (46 h) every 2 weeks vs. GemCIs: cisplatin 25 mg/m^2^ followed by Gem 1000 mg/m^2^ (on days 1 and 8) every 3 weeks	n.a	n.a
**Berger et al. [30] Phase II NIFE trial**	locally advanced, non-resectable or metastatic BTC	Nal-IRI: nal-IRI mg/m^2^ (46 h infusion), 5-FU 2400 mg/m^2^ (46 h infusion), leucovorin 400 mg/m^2^ (0.5 h infusion) on day 1 every 2 weeks Vs. GemCis: cisplatin 25 mg/m^2^ followed by Gem 1000 mg/m^2^ (on days 1 and 8) every 3 weeks	n.a	n.a

CCA = cholangiocarcinoma; BTC = biliary tract cancer; Gem = gemcitabine; Cap = capecitabine; Cis = cisplatin; Oxali = oxaliplatin; IRI = irinotecan; 5-FU = 5-fluorouracil; nal = nanoliposomial; GemOx = gemcitabine, oxaliplatin; OS = overall survival; n.a = not available; PFS = progression free survival; CI = confidence.

**Table 3:** Summary of treatments in advanced settings.

	After First-Line			
Study	Number and Type of Patients	Study Arms	PFS	OS
**Lamarca et al. [21] randomised phase III, multi-centre, open-label ABC-06 trial**	162 locally advanced/metastatic BTC patients previously-treated with CisGem chemotherapy	ASC+ mFOLFOX6: Oxali 85 mg/m^2^, folinic acid 350 mg/m^2^, 5-FU 400 mg/m^2^ (bolus) and 2400 mg/m^2^ (infusion) every 2 weeks for 12 cycles vs. ASC		mOS: 6.2 vs. 5.3 months HR: 0.69 (95% CI 0.50–0.97; *p* = 0.031) 6-months OS: 50.6% vs. 35.5% 12-months OS: 25.9% vs. 11.4%
**Zheng et al. [33] randomised, Phase II study**	64 advanced BTC patients progressed after CisGem	XELIRI: IRI 180 mg/m^2^ on day 1, cap 1000 mg/m^2^ twice daily on days 1–10 every 2 weeks vs. Irinotecan: IRI 180 mg/m^2^ every 2 weeks	3.7 vs. 2.4 months	9-month OS: 60.9% vs. 32.0%, mOS 10.1 vs. 7.3 months DCR: 63.3% vs. 50.0%
**Guion-Dusserre et al. [34]**	13 metastatic intrahepatic CCA patients who were refractory to GemOx	FOLFIRI + bevacizumab: IRI 180 mg/m^2^ (IV 90 min), folinic acid 400 mg/m^2^ (IV 2h), 5-FU 2400 mg/m^2^ (46h), bevacizumab (5 mg/kg) every 2 weeks	8 months (95% CI: 7–16 months)	20 months (95% CI: 8–48 months) DCR: 84.5% (95% CI: 42%–100%) ORR: 38.4% (95% CI: 12.5%–89%)
**Sebbagh et al. [35] retrospective trial**	52 recurrent/advanced BTC patients after progression to GemOx	FOLFIRI (after GemOx in fist line): IRI 180 mg/m^2^ (IV 90 min), folinic acid 400 mg/m^2^ (IV 2h), 5-FU 2400 mg/m^2^ (46h)	Fist-line GemOx: 4.8 months Second-line FOLFIRI: 3.2 months	21.9 months
**Chakrabarti et al. [36] Phase II trial**	27 advanced BTC previously treated	FTD/TPI 35 mg/m^2^/day	3.8 months (95% CI, 2.0 to 5.8 months)	6.1 months (95% CI, 4.4 to 11.4 months)

CCA = cholangiocarcinoma; BTC = biliary tract cancer; Gem = gemcitabine; CisGem = cisplatin, gemcitabine; Cap = capecitabine; obs = observation; Cis = cisplatin; Oxali = oxaliplatin; IRI = irinotecan; 5-FU = 5-fluorouracil; GemOx = gemcitabine, oxaliplatin; FTD/TPI = trifluridine/tipiracil; ASC = active symptom control; OS = overall survival; TTP = time to progression; PFS = progression free survival; DCR = disease control rate; ORR = objective response rate; CI = confidence interval.
